# Supplementary material for: Xerophilic Aspergillaceae Dominate the Communities of Culturable Fungi in the Mound Nests of the Western Thatching Ant (Formica obscuripes)
Source: J Fungi (Basel). 2024 Oct 23;10(11):735. doi: 10.3390/jof10110735 (PMC11595882; doi:10.3390/jof10110735)
Supplement: Supplementary file 1 [file jof-10-00735-s001.zip › Supplementary Table S4. Isolates used in assessments of xerotolerance.pdf]

Table S4. Accession numbers of isolates used in studies of xerotolerance.

Taxa	Strain numbers	UAMH accession numbers	GenBank Accession numbers		
			ITS	*CaM*	*TUB*
Aspergillus europaeus	Asp 4-1; M1-4G	UAMH 12242	OP456343	-	OP676268
	Asp 4-3; M2-4G	-	-	-	-
	Asp 4-2; M2-3G	-	-	-	-
Aspergillus fructus	Asp 3-2; M2-4RB	UAMH 12241	OP456342	OP676286	OP676267
	Asp 3-3; M3-4G	-	-	-	-
	Asp 3-1; M1-4G	-	-	-	-
Aspergillus insuetus	Asp 2-2; M2-4RB	UAMH 12240	OP456341	-	OP676266
	Asp 2-3: M3-4G	-	-	-	-
	Asp 2-1; M1-4G	-	-	-	-
Aspergillus tubingensis	Asp 1-2; M2-4G	UAMH 12239	OP456340	OP676285	OP676265
	Asp 1-3; M3-4RB	-	-	-	-
	Asp 1-1; M1-3G	-	-	-	-
Penicillium charlesii	Pen 9-3; M3-4S	UAMH 12232	OP456353	-	OP676278
	Pen 9-1; L1-4G	-	-	-	-
	Pen 9-2: M2-4G	-	-	-	-
Penicillium chrysogenum	Pen 15-3; M1-5S	UAMH 12237	OP456358	-	OP676283
	Pen 15-2; M1-5S	-	-	-	-
	Pen 15-1; M1-5S	-	-	-	-
Penicillium citrinum	Pen 5-1; M1-4G	UAMH 12227	OP456348	-	OP676273
	Pen 5-3; N2-4S	-	-	-	-
	Pen 5-2; L2-4RB	-	-	-	-
Penicillium estinogenum	Pen 12-2; N1-3G	UAMH 12235	OP456356	-	OP676281
	Pen 12-3; N1-3G	-	-	-	-
	Pen 12-1; N1-3G	-	-	-	-
Penicillium parvulum	Pen 11-1; M1-4RB	UAMH 12234	OP456355	-	OP676280
	Pen 11-3; L3-4RB	-	-	-	-
	Pen 11-2; M2-4G	-	-	-	-
Penicillium pasqualense	Pen 4-3; N3-3G	UAMH 12226	OP456347	-	OP676272
	Pen 4-1; L3-4RB	-	-	-	-
	Pen 4-2; N2-4S	-	-	-	-
Penicillium sanguifluum	Pen 7b; L2-5RB	UAMH 12230	OP456351	OP676289	OP676276
Penicillium scabrosum	Pen 6-1; N1-3S	UAMH 12228	OP456349	OP676287	OP676274
	Pen 6-3; N3-3G	-	-	-	-
	Pen 6-2; L2-4S	-	-	-	-
Penicillium sizovae	Pen 14-2; L3-5S	UAMH 12236	OP456357	-	OP676282
	Pen 14-3; L3-4G	-	-	-	-

Taxa	Strain numbers	UAMH accession numbers	GenBank Accession numbers		
			ITS	*CaM*	*TUB*
	Pen 14-1; L1-4S	-		-	
Penicillium skrjabinii	Pen 3-3; N3-4RB	UAMH 12225	OP456346	-	OP676271
	Pen 3-1; N1-3S	-	-	-	-
	Pen 3-2; N2-46	-	-	-	-
Penicillium soppii	Pen 1-1; N1-3S	UAMH 12223	OP456344	-	OP676269
	Pen 1-3; N2-3G	-	-	-	-
	Pen 1-2; N2-3G	-	-	-	-
Penicillium thomii	Pen 2-1; M2-6RB	UAMH 12224	OP456345	-	OP676270
	Pen 2-2; M2-6RB	-	-	-	-
Penicillium turbatum	Pen 8-2; L1-5G	UAMH 12231	OP456352	-	OP676277
	Pen 8-3; M3-4S	-	-	-	-
	Pen 8-1; M1-4RB	-	-	-	-
Penicillium yarmokense	Pen 7-1; N1-3S	UAMH 12229	OP456350	OP676288	OP676275
	Pen 7-3; N3-3G	-	-	-	-
	Pen 7-2; N3-3G	-	-	-	-
Pseudogymnoascus pannorum 1	Pp1 (1-2); M2-4G	UAMH 12243	OP456360	-	-
	Pp1 (1-1); N1-3G	-	-	-	-
	Pp1 (1-3); L3-4	-	-	-	-
Pseudogymnoascus pannorum 2	Pp2 (2-1); L1-5G	UAMH 12244	OP456361	-	-
	Pp2 (2-1); M1-4RB	-	-	-	-
	Pp2 (2-3); M3-4RB	-	-	-	-
Talaromyces atricola	Pen 17-1; M1-4RB	UAMH 12238	OP456359	-	OP676284
	Pen 17-2; M3-4G	-	-	-	-
	Pen 17-3: M1-4S	-	-	-	-
Talaromyces neorugulosus	Pen 10-3; M3-4RB	UAMH 12233	OP456354	-	OP676279
	Pen 10-1; L3-5G	-	-	-	-
	Pen 10-2; M2-4S	-	-	-	-
