# Supplementary material for: Xerophilic Aspergillaceae Dominate the Communities of Culturable Fungi in the Mound Nests of the Western Thatching Ant (Formica obscuripes)
Source: J Fungi (Basel). 2024 Oct 23;10(11):735. doi: 10.3390/jof10110735 (PMC11595882; doi:10.3390/jof10110735)
Supplement: Supplementary file 1 [file jof-10-00735-s001.zip › Supplementary Table S5. Growth rates.pdf]

Table S5. Mean growth rates (mm d^{-1}) and coefficients of determination (R^2) of isolates of *Aspergillus*, *Penicillium*, *Pseudogymnoascus* and *Talaromyces* on MYA containing different concentrations of glycerol and sucrose. The number of isolates examined appears in brackets after the name of each taxon.

Osmoticant	Concentration (mol L^{-1})	a_w	*Aspergillus europaeus* (3)		*Aspergillus fructus* (3)		*Aspergillus insuetus* (3)	
			Growth rate	R^2	Growth rate	R^2	Growth rate	R^2
MYA alone	0	1.00	1.25	0.994	1.31	0.999	2.12	0.996
glycerol	1.0	0.97	3.53	0.996	2.04	0.998	2.76	0.999
	2.0	0.95	3.70	0.992	1.88	0.996	1.96	0.998
	3.0	0.92	3.15	0.998	1.15	0.997	1.05	0.999
	4.0	0.89	2.27	0.971	0.66	0.993	0.45	0.993
	5.0	0.84	0.40	0.904	0.30	0.973	0.12	0.973
	6.0	0.80	0	-	0	-	0	-
	7.0	0.73	0	-	0	-	0	-
sucrose	0.5	0.99	4.16	0.995	1.77	0.996	3.37	0.998
	1.0	0.97	4.99	0.987	2.53	0.999	2.64	0.999
	1.5	0.94	4.89	0.992	2.23	0.999	1.60	0.999
	2.0	0.90	3.15	0.995	1.19	0.999	0.60	0.996
	2.5	0.78	0.61	0.976	0.20	0.928	0.07	0.927
	3.0	0.74	0	-	0	-	0	-

Osmoticant	Concentration (mol/L)	a_w	*Aspergillus tubingensis* (3)		*Penicillium charlesii* (3)		*Penicillium chrysogenum* (3)	
			Growth rate	R^2	Growth rate	R^2	Growth rate	R^2
MYA alone	0	1.00	5.50	0.999	0.86	0.980	2.59	0.999
glycerol	1.0	0.97	6.50	0.994	1.42	0.985	3.71	0.994
	2.0	0.95	4.67	0.998	1.37	0.991	2.91	0.994
	3.0	0.92	3.50	0.999	1.04	0.997	2.06	0.999
	4.0	0.89	2.13	0.995	0.50	0.995	1.00	0.999
	5.0	0.84	0.77	0.968	0.13	0.961	0.45	0.971
	6.0	0.80	0	-	0	-	0	-
	7.0	0.73	0	-	0	-	0	-
sucrose	0.5	0.99	7.06	0.999	1.12	0.987	4.31	0.980
	1.0	0.97	6.59	0.999	1.65	0.998	3.80	0.994
	1.5	0.94	5.06	0.998	1.40	0.998	2.56	0.998
	2.0	0.90	2.29	0.999	0.67	0.983	1.24	0.998
	2.5	0.78	0.32	0.886	0.08	0.903	0.19	0.975
	3.0	0.74	0	-	0	-	0	-

Osmoticant	Concentration (mol/L)	a_w	*Penicillium citrinum* (3)		*Penicillium estinogenum* (3)		*Penicillium parvulum* (3)	
			Growth rate	R^2	Growth rate	R^2	Growth rate	R^2
MYA alone	0	1.00	1.98	0.997	2.01	0.980	0.81	0.971
glycerol	1.0	0.97	2.39	0.989	2.50	0.998	1.36	0.996
	2.0	0.95	2.13	0.998	2.02	0.999	1.37	0.999
	3.0	0.92	1.30	0.993	1.12	0.997	1.13	0.999
	4.0	0.89	0.61	0.983	0.38	0.977	0.68	0.997
	5.0	0.84	0.28	0.990	0.05	0.889	0.30	0.949
	6.0	0.80	0	-	0	-	0	-
	7.0	0.73	0	-	0	-	0	-
sucrose	0.5	0.99	2.54	0.987	2.90	0.992	1.27	0.999
	1.0	0.97	2.95	0.999	2.81	0.997	1.36	0.997
	1.5	0.94	2.30	0.999	1.92	0.999	1.24	0.999
	2.0	0.90	1.28	0.997	0.57	0.977	0.67	0.992
	2.5	0.78	0.31	0.960	0	-	0.09	0.924
	3.0	0.74	0	-	0	-	0	-

Osmoticant	Concentration (mol/L)	a_w	*Penicillium pasqualense* (3)		*Penicillium sanguifluum* (1)		*Penicillium scabrosum* (3)	
			Growth rate	R^2	Growth rate	R^2	Growth rate	R^2
MYA alone	0	1.00	1.44	0.996	1.41	0.994	1.55	0.997
glycerol	1.0	0.97	1.78	0.984	1.66	0.994	2.30	0.999
	2.0	0.95	1.35	0.996	1.48	0.987	2.16	0.999
	3.0	0.92	1.02	0.995	0.98	0.978	1.45	0.986
	4.0	0.89	0.48	0.997	0.50	0.975	0.59	0.995
	5.0	0.84	0.16	0.977	0.17	0.969	0.13	0.977
	6.0	0.80	0	-	0	-	0	-
	7.0	0.73	0	-	0	-	0	-
sucrose	0.5	0.99	1.82	0.990	1.70	0.995	2.15	0.998
	1.0	0.97	1.60	0.992	1.98	0.998	2.61	0.999
	1.5	0.94	1.41	0.996	1.48	0.996	2.21	0.999
	2.0	0.90	0.56	0.982	0.72	0.971	1.02	0.994
	2.5	0.78	0.08	0.927	0.11	0.924	0.12	0.927
	3.0	0.74	0	-	0	-	0	-

Osmoticant	Concentration (mol/L)	a_w	***Penicillium sizovae*** **(3)**		***Penicillium skrjabinii*** **(3)**		***Penicillium soppii*** **(3)**	
			**Growth rate**	R^2	**Growth rate**	R^2	**Growth rate**	R^2
MYA alone	0	1.00	1.62	0.993	2.90	0.999	2.16	0.991
glycerol	1.0	0.97	2.52	0.998	3.12	0.999	2.88	0.991
	2.0	0.95	2.01	0.998	2.25	0.997	2.35	0.994
	3.0	0.92	1.12	0.982	1.12	0.999	1.52	0.994
	4.0	0.89	0.54	0.994	0.34	0.978	0.57	0.995
	5.0	0.84	0.24	0.978	0.05	0.893	0.18	0.963
	6.0	0.80	0	-	0	-	0	-
	7.0	0.73	0	-	0	-	0	-
sucrose	0.5	0.99	2.80	0.994	3.48	0.998	3.12	0.988
	1.0	0.97	2.80	0.994	2.52	0.992	3.08	0.989
	1.5	0.94	2.00	0.994	1.47	0.992	2.19	0.978
	2.0	0.90	0.99	0.998	0.48	0.989	0.60	0.980
	2.5	0.78	0.14	0.902	0	-	0.09	0.920
	3.0	0.74	0	-	0	-	0	-

Osmoticant	Concentration (mol/L)	a_w	***Penicillium thomii*** **(2)**		***Penicillium turbatum*** **(3)**		***Penicillium yarmokense*** **(3)**	
			**Growth rate**	R^2	**Growth rate**	R^2	**Growth rate**	R^2
MYA alone	0	1.00	1.66	0.985	1.53	0.986	1.23	0.991
glycerol	1.0	0.97	3.93	0.991	2.27	0.999	1.33	0.995
	2.0	0.95	2.78	0.995	1.84	0.999	0.94	0.995
	3.0	0.92	1.81	0.995	1.27	0.996	0.74	0.998
	4.0	0.89	0.45	0.993	0.50	0.994	0.55	0.964
	5.0	0.84	0.02	0.973	0.05	0.975	0.17	0.979
	6.0	0.80	0	-	0	-	0	-
	7.0	0.73	0	-	0	-	0	-
sucrose	0.5	0.99	4.73	0.996	2.24	0.999	1.80	0.994
	1.0	0.97	3.65	0.996	1.77	0.994	1.67	0.966
	1.5	0.94	2.16	0.984	1.11	0.998	1.26	0.991
	2.0	0.90	0.56	0.979	0.57	0.982	0.65	0.999
	2.5	0.78	0.02	0.874	0.05	0.903	0.11	0.969
	3.0	0.74	0	-	0	-	0	-

Osmoticant	Concentration (mol/L)	a_w	***Pseud. pannorum* 1 (3)**		***Pseud. pannorum* 2 (3)**		***Talaromyces atricola* (3)**	
			**Growth rate**	***R^2***	**Growth rate**	***R^2***	**Growth rate**	***R^2***
MYA alone	0	1.00	0.36	0.961	0.71	0.997	0.85	0.991
glycerol	1.0	0.97	0.46	0.982	0.80	0.990	0.91	0.996
	2.0	0.95	0.27	0.995	0.43	0.988	0.86	0.989
	3.0	0.92	0.07	0.951	0.11	0.978	0.33	0.969
	4.0	0.89	0	-	0	-	0.04	0.890
	5.0	0.84	0	-	0	-	0	-
	6.0	0.80	0	-	0	-	0	-
	7.0	0.73	0	-	0	-	0	-
sucrose	0.5	0.99	0.43	0.976	1.04	0.985	0.86	0.993
	1.0	0.97	0.39	0.991	0.93	0.987	0.93	0.997
	1.5	0.94	0.18	0.995	0.51	0.990	0.72	0.993
	2.0	0.90	0	-	0.07	0.909	0.29	0.974
	2.5	0.78	0	-	0	-	0	-
	3.0	0.74	0	-	0	-	0	-

Osmoticant	Concentration (mol/L)	a_w	***Talaromyces neorugulosus* (3)**	
			**Growth rate**	***R^2***
MYA alone	0	1.00	0.92	0.989
glycerol	1.0	0.97	1.16	0.992
	2.0	0.95	0.92	0.991
	3.0	0.92	0.53	0.984
	4.0	0.89	0.11	0.979
	5.0	0.84	0	-
	6.0	0.80	0	-
	7.0	0.73	0	-
sucrose	0.5	0.99	1.38	0.999
	1.0	0.97	1.20	0.997
	1.5	0.94	0.80	0.997
	2.0	0.90	0.29	0.975
	2.5	0.78	0	-
	3.0	0.74	0	-
