# Supplementary material for: Xerophilic Aspergillaceae Dominate the Communities of Culturable Fungi in the Mound Nests of the Western Thatching Ant (Formica obscuripes)
Source: J Fungi (Basel). 2024 Oct 23;10(11):735. doi: 10.3390/jof10110735 (PMC11595882; doi:10.3390/jof10110735)
Supplement: Supplementary file 1 [file jof-10-00735-s001.zip › Supplementary Table S6. Statistics.pdf]

Table S6. Permutational multivariate analysis of variance (PERMANOVA) of fungal communities from soils from the tops of mound nests (M), within nests (L) and non-mound (N) sites.

**Pairwise comparison**	**F-statistic**	***P*-value ***
M - L	4.0480	0.0003
M - N	7.2046	0.0037
L - N	8.5380	0.0003

* *P*-values have been adjusted using the B-H correction.
