# Supplementary material for: Xerophilic Aspergillaceae Dominate the Communities of Culturable Fungi in the Mound Nests of the Western Thatching Ant (Formica obscuripes)
Source: J Fungi (Basel). 2024 Oct 23;10(11):735. doi: 10.3390/jof10110735 (PMC11595882; doi:10.3390/jof10110735)
Supplement: Supplementary file 1 [file jof-10-00735-s001.zip › Supplementary Tables S1-S3. DRBA, DRBAG and MEA20S.pdf]

Table S1. Relative abundance (RA) and colony forming units per gram of soil (CFU g^{-1}) of Fungi recovered on Dichloran Rose Bengal agar (DRBA) from soils from the tops of mound nests (M), within mound nests (L), and adjacent non-mound (N) sites.

Taxon	M1		M2		M3		L1		L2		L3		N1		N2		N3		Total
	RA	CFU g^{-1}	RA	CFU g^{-1}	RA	CFU g^{-1}	RA	CFU g^{-1}	RA	CFU g^{-1}	RA	CFU g^{-1}	RA	CFU g^{-1}	RA	CFU g^{-1}	RA	CFU g^{-1}	CFU g^{-1}
Acremonium 05	-	-	-	-	1.92	2.24E+04	-	-	-	-	-	-	-	-	-	-	-	-	2.24E+04
Acremonium 06	1.69	2.26E+05	-	-	-	-	-	-	-	-	-	-	-	-	-	-	-	-	2.26E+05
Acremonium 08	-	-	-	-	-	-	-	-	-	-	-	-	-	-	-	-	-	-	0.00E+00
Acremonium 10	-	-	2.38	2.23E+04	-	-	-	-	-	-	-	-	-	-	-	-	-	-	2.23E+04
Acremonium 11	1.69	2.26E+04	-	-	1.92	2.24E+04	1.75	2.26E+05	-	-	-	-	-	-	-	-	-	-	2.71E+05
Acremonium 13	-	-	-	-	-	-	1.75	2.26E+05	-	-	-	-	-	-	-	-	-	-	2.26E+05
Acremonium 14	-	-	-	-	-	-	1.75	2.26E+04	3.23	2.31E+05	1.96	2.33E+04	-	-	-	-	-	-	2.77E+05
Acremonium 15	-	-	-	-	-	-	1.75	2.26E+04	3.23	2.31E+04	-	-	-	-	-	-	-	-	4.57E+04
Albifimbria verrucaria	1.69	2.26E+04	-	-	-	-	1.75	2.26E+04	-	-	-	-	-	-	-	-	-	-	4.51E+04
Aspergillus fructus	10.17	1.35E+05	19.05	1.79E+05	3.85	4.49E+04	19.30	2.48E+05	9.68	6.94E+04	15.69	1.86E+05	11.11	2.43E+05	-	-	-	-	1.11E+06
Aspergillus insuetus	1.69	2.26E+04	7.14	6.70E+04	1.92	2.24E+04	1.75	2.26E+05	6.45	4.62E+04	3.92	4.66E+04	-	-	-	-	-	-	4.31E+05
Aspergillus tubingensis	1.69	2.26E+05	4.76	4.47E+04	9.62	1.12E+05	5.26	6.77E+04	9.68	6.94E+04	3.92	4.66E+04	-	-	-	-	-	-	5.66E+05
Aureobasidium 01	-	-	-	-	1.92	2.24E+04	-	-	-	-	-	-	-	-	-	-	9.09	2.53E+04	4.78E+04
Aureobasidium 02	-	-	2.38	2.23E+04	1.92	2.24E+04	-	-	-	-	-	-	-	-	-	-	-	-	4.48E+04
Auxarthron californiense	-	-	-	-	-	-	-	-	-	-	-	-	-	-	6.67	2.47E+04	-	-	2.47E+04
Basifimbria sp.	-	-	-	-	-	-	-	-	-	-	1.96	2.33E+04	-	-	-	-	-	-	2.33E+04
Chrysosporium 02	-	-	-	-	-	-	-	-	-	-	3.92	4.66E+04	-	-	-	-	-	-	4.66E+04
Cepahlotrichum microsporum	1.69	2.26E+05	-	-	-	-	-	-	3.23	2.31E+04	3.92	4.66E+04	-	-	-	-	-	-	2.95E+05
Cephalotrichum nanum	-	-	-	-	-	-	-	-	3.23	2.31E+04	-	-	-	-	-	-	-	-	2.31E+04
Cladosporium cladosporioides	1.69	2.26E+04	2.38	2.23E+07	1.92	2.24E+04	-	-	-	-	-	-	-	-	-	-	-	-	2.24E+07
Cladosporium herbarum	5.08	6.77E+04	2.38	2.23E+04	3.85	4.49E+04	1.75	2.26E+04	-	-	-	-	-	-	-	-	-	-	1.58E+05
Cladosporium cf. *macrocarpum*	1.69	2.26E+04	-	-	-	-	-	-	-	-	-	-	-	-	-	-	-	-	2.26E+04
Cladosporium (ungrouped)	-	-	2.38	2.23E+04	-	-	-	-	-	-	-	-	-	-	6.67	2.47E+04	-	-	4.70E+04
Clonostachys rosea	-	-	-	-	-	-	-	-	-	-	-	-	-	-	-	-	9.09	2.53E+04	2.53E+04
Exophiala sp.	1.69	2.26E+04	-	-	1.92	2.24E+04	-	-	-	-	-	-	-	-	13.33	4.93E+04	9.09	2.53E+04	1.20E+05
Fusarium 01	-	-	-	-	-	-	1.75	2.26E+05	-	-	-	-	-	-	6.67	2.47E+04	-	-	2.50E+05
Fusarium 08	-	-	-	-	-	-	-	-	-	-	-	-	-	-	6.67	2.47E+04	-	-	2.47E+04
Fusarium 11	-	-	-	-	-	-	-	-	-	-	-	-	-	-	-	-	9.09	2.53E+04	2.53E+04
Geotrichum 02	-	-	-	-	-	-	1.75	2.26E+04	-	-	-	-	-	-	-	-	-	-	2.26E+04
Leptosphaeria coniothyrium	-	-	-	-	1.92	2.24E+05	-	-	-	-	-	-	-	-	-	-	-	-	2.24E+05
Marquandomyces marquandii	-	-	2.38	2.23E+04	1.92	2.24E+04	1.75	2.26E+05	3.23	2.31E+04	1.96	2.33E+04	11.11	2.43E+03	6.67	2.47E+04	9.09	2.53E+04	3.69E+05
Microascus sp.	1.69	2.26E+05	-	-	-	-	1.75	2.26E+04	-	-	1.96	2.33E+05	-	-	-	-	-	-	4.81E+05
Microascus paisii	1.69	2.26E+04	7.14	6.70E+04	1.92	2.24E+06	1.75	2.26E+04	-	-	3.92	4.66E+04	-	-	-	-	-	-	2.40E+06
Penicillium charlesii	1.69	2.26E+04	9.52	8.94E+04	-	-	1.75	2.26E+04	-	-	1.96	2.33E+04	-	-	-	-	-	-	1.58E+05
Penicillium citrinum	5.08	6.77E+04	7.14	6.70E+04	9.62	1.12E+05	36.84	4.74E+05	25.81	1.85E+05	3.92	4.66E+04	11.11	2.43E+05	6.67	2.47E+05	9.09	2.53E+04	1.47E+06
Penicillium parvulum	1.69	2.26E+04	-	-	1.92	2.24E+04	1.75	2.26E+04	-	-	1.96	2.33E+04	-	-	-	-	-	-	9.09E+04

Taxon	M1		M2		M3		L1		L2		L3		N1		N2		N3		Total
	RA	CFU g^{-1}	RA	CFU g^{-1}	RA	CFU g^{-1}	RA	CFU g^{-1}	RA	CFU g^{-1}	RA	CFU g^{-1}	RA	CFU g^{-1}	RA	CFU g^{-1}	RA	CFU g^{-1}	CFU g^{-1}
Penicillium pasqualense	1.69	2.26E+04	-	-	1.92	2.24E+06	-	-	12.90	9.25E+04	3.92	4.66E+04	11.11	2.43E+05	6.67	2.47E+04	-	-	2.67E+06
Penicillium sanguifluum	-	-	-	-	-	-	-	-	3.23	2.31E+05	-	-	-	-	-	-	-	-	2.31E+05
Penicillium scabrosum	1.69	2.26E+04	-	-	-	-	-	-	-	-	-	-	-	-	6.67	2.47E+04	-	-	4.72E+04
Penicillium sizovae	-	-	-	-	-	-	-	-	-	-	7.84	9.32E+04	-	-	-	-	-	-	9.32E+04
Penicillium skrjabinii	1.69	2.26E+04	-	-	-	-	-	-	-	-	-	-	-	-	-	-	9.09	2.53E+04	4.79E+04
Penicillium soppii	1.69	2.26E+05	-	-	-	-	-	-	-	-	-	-	-	-	-	-	-	-	2.26E+05
Penicillium thomii	-	-	2.38	2.23E+06	-	-	-	-	-	-	-	-	-	-	-	-	-	-	2.23E+06
Penicillium turbatum	3.39	4.51E+04	-	-	11.54	1.35E+05	-	-	-	-	-	-	44.44	9.72E+04	-	-	-	-	2.77E+05
Penicillium yarmokense	1.69	2.26E+04	-	-	-	-	-	-	3.23	2.31E+04	-	-	-	-	6.67	2.47E+04	-	-	7.04E+04
Penicillium (ungrouped)	1.69	2.26E+04	-	-	-	-	1.75	2.26E+05	3.23	2.31E+04	-	-	-	-	-	-	-	-	2.71E+05
Pseudogymnoascus pannorum 1	20.34	2.71E+05	9.52	8.94E+04	13.46	1.57E+05	3.51	4.51E+04	6.45	4.62E+04	29.41	3.50E+05	-	-	-	-	-	-	9.58E+05
Pseudo. pannorum 2	5.08	6.77E+04	4.76	4.47E+04	3.85	4.49E+04	1.75	2.26E+05	3.23	2.31E+04	1.96	2.33E+04	11.11	2.43E+05	-	-	-	-	6.72E+05
Purpureocillium lilacinum	-	-	-	-	1.92	2.24E+05	1.75	2.26E+04	-	-	1.96	2.33E+04	-	-	6.67	2.47E+04	9.09	2.53E+04	3.20E+05
Pycnidia 01	1.69	2.26E+05	-	-	1.92	2.24E+04	-	-	-	-	-	-	-	-	-	-	9.09	2.53E+04	2.73E+05
Pycnidia 03	-	-	-	-	1.92	2.24E+04	-	-	-	-	-	-	-	-	-	-	-	-	2.24E+04
Pycnidia 04	-	-	2.38	2.23E+04	1.92	2.24E+04	-	-	-	-	-	-	-	-	-	-	-	-	4.48E+04
Pycnidia 05	1.69	2.26E+05	2.38	2.23E+04	-	-	-	-	-	-	-	-	-	-	-	-	-	-	2.48E+05
Scopulariopsis candida	1.69	2.26E+04	-	-	-	-	-	-	-	-	-	-	-	-	-	-	-	-	2.26E+04
Talaromyces atricola	1.69	2.26E+04	-	-	-	-	-	-	-	-	-	-	-	-	-	-	-	-	2.26E+04
Talaromyces neorugulosus	-	-	2.38	2.23E+04	1.92	2.24E+04	-	-	-	-	1.96	2.33E+05	-	-	-	-	-	-	2.78E+05
Tolypocladium inflatum	-	-	-	-	-	-	1.75	2.26E+04	-	-	1.96	2.33E+04	-	-	-	-	-	-	4.59E+04
Trichoderma sp.	-	-	-	-	-	-	-	-	-	-	-	-	-	-	6.67	2.47E+04	-	-	2.47E+04
Trichosporiella cerebriformis	-	-	2.38	2.23E+04	-	-	-	-	-	-	-	-	-	-	-	-	-	-	2.23E+04
Volutella ciliata	-	-	-	-	-	-	-	-	-	-	-	-	-	-	-	-	9.09	2.53E+04	2.53E+04
Sterile 01	-	-	-	-	-	-	-	-	-	-	-	-	-	-	6.67	2.47E+04	-	-	2.47E+04
Sterile 03	-	-	2.38	2.23E+04	-	-	-	-	-	-	-	-	-	-	-	-	-	-	2.23E+04
Sterile 04	1.69	2.26E+05	-	-	1.92	2.24E+04	-	-	-	-	-	-	-	-	-	-	-	-	2.48E+05
Sterile 05	1.69	2.26E+04	-	-	1.92	2.24E+06	1.75	2.26E+04	-	-	-	-	-	-	-	-	-	-	2.29E+06
Sterile (ungrouped)	6.78	9.03E+04	2.38	2.23E+04	5.77	6.73E+04	1.75	2.26E+05	-	-	-	-	-	-	6.67	2.47E+04	9.09	2.53E+05	6.84E+05
Sporulating but undetermined	1.69	2.26E+04	-	-	1.92	2.24E+04	-	-	-	-	-	-	-	-	-	-	-	-	4.50E+04
Total	100.0	**2.96E+06**	100.0	**2.55E+07**	100.0	**8.24E+06**	100.0	2.91E+06	100.0	1.13E+06	100.0	1.61E+06	100.0	1.07E+06	100.0	5.92E+05	100.0	5.06E+05	4.45E+07
Total Aspergillaceae	35.6	8.80E+05	50.0	2.68E+06	40.4	2.69E+06	68.4	1.29E+06	74.2	7.40E+05	43.1	5.13E+05	77.8	8.26E+05	26.7	3.21E+05	18.2	5.06E+04	9.99E+06
Total *Aspergillus*	13.6	3.84E+05	31.0	2.91E+05	15.4	1.80E+05	26.3	5.42E+05	25.8	1.85E+05	23.5	2.80E+05	11.1	2.43E+05	-	-	-	-	2.10E+06
Total *Penicillium*	22.0	4.97E+05	19.0	2.39E+06	25.0	2.51E+06	42.1	7.45E+05	48.4	5.55E+05	19.6	2.33E+05	66.7	5.83E+05	26.7	3.21E+05	18.2	5.06E+04	7.89E+06
Total *Talaromyces*	1.7	2.26E+04	2.4	2.23E+04	1.9	2.24E+04	-	-	-	-	2.0	2.33E+05	-	-	-	-	-	-	3.00E+05
Total *Pseudogymnoascus*	25.4	3.39E+05	14.3	1.34E+05	17.3	2.02E+05	5.3	2.71E+05	9.7	6.94E+04	31.4	3.73E+05	11.1	2.43E+05	-	-	-	-	1.63E+06
Total Chaetothyriales	1.7	2.26E+04	-	-	1.9	2.24E+04	-	-	-	-	-	-	-	-	13.3	4.93E+04	9.1	2.53E+04	1.20E+05

Taxon	M1		M2		M3		L1		L2		L3		N1		N2		N3		Total
	RA	CFU g^{-1}	RA	CFU g^{-1}	RA	CFU g^{-1}	RA	CFU g^{-1}	RA	CFU g^{-1}	RA	CFU g^{-1}	RA	CFU g^{-1}	RA	CFU g^{-1}	RA	CFU g^{-1}	CFU g^{-1}
Total Cladosporiales	8.5	1.13E+05	7.1	2.24E+07	5.8	6.73E+04	1.8	2.26E+04	-	-	-	-	-	-	6.7	2.47E+04	-	-	2.26E+07
Total Hypocreales	5.1	2.71E+05	4.8	4.47E+04	7.7	2.92E+05	15.8	1.02E+06	9.7	2.77E+05	7.8	9.32E+04	11.1	2.43E+03	33.3	1.23E+05	45.5	1.27E+05	2.25E+06
Total Microascales	6.8	4.97E+05	7.1	6.70E+04	1.9	2.24E+06	3.5	4.51E+04	6.5	4.62E+04	9.8	3.26E+05	-	-	-	-	-	-	3.23E+06
Total Onygenales	-	-	-	-	-	-	-	-	-	-	3.9	4.66E+04	-	-	6.7	2.47E+04	-	-	7.13E+04
Total sterile and undetermined	15.3	8.13E+05	9.5	8.94E+04	17.3	2.42E+06	3.5	2.48E+05	-	-	-	-	-	-	13.3	4.93E+04	18.2	2.78E+05	3.90E+06

Table S2. Relative abundance (RA) and colony forming units per gram of soil (CFU g^{-1}) of Fungi recovered on Dichloran Rose Bengal agar containing 18% glycerol (DRBAG) from soils from the tops of mound nests (M), within mound nests (L), and adjacent non-mound (N) sites.

Taxon	M1		M2		M3		L1		L2		L3		N1		N2		N3		Total
	RA	CFU g^{-1}	RA	CFU g^{-1}	RA	CFU g^{-1}	RA	CFU g^{-1}	RA	CFU g^{-1}	RA	CFU g^{-1}	RA	CFU g^{-1}	RA	CFU g^{-1}	RA	CFU g^{-1}	CFU g^{-1}
Acremonium 02	-	-	-	-	-	-	-	-	-	-	-	-	-	-	2.63	2.47E+03	-	-	2.47E+03
Acremonium 04	-	-	-	-	-	-	-	-	-	-	-	-	1.72	2.43E+03	2.63	2.47E+03	3.85	2.53E+03	7.43E+03
Acremonium 08	-	-	-	-	-	-	-	-	-	-	-	-	-	-	2.63	2.47E+03	-	-	2.47E+03
Acremonium 09	-	-	-	-	-	-	-	-	-	-	-	-	1.72	2.43E+04	-	-	-	-	2.43E+04
Acremonium 11	-	-	-	-	0.61	2.24E+03	-	-	-	-	-	-	-	-	-	-	-	-	2.24E+03
Acremonium 14	-	-	-	-	-	-	-	-	-	-	2.56	2.33E+05	-	-	2.63	2.47E+04	-	-	2.58E+05
Acremonium 15	-	-	-	-	-	-	1.72	2.26E+04	-	-	-	-	-	-	-	-	-	-	2.26E+04
Alternaria (ungrouped)	-	-	0.83	2.23E+05	-	-	-	-	-	-	-	-	-	-	-	-	-	-	2.23E+05
Aspergillus europaeus	0.95	2.26E+04	0.83	2.23E+04	-	-	-	-	-	-	-	-	-	-	-	-	3.85	2.53E+03	4.75E+04
Aspergillus fructus	14.29	3.39E+04	5.00	1.34E+05	1.21	4.49E+03	15.52	2.03E+05	25.00	2.31E+04	25.64	2.33E+05	3.45	4.86E+04	5.26	4.93E+03	3.85	2.53E+03	6.88E+05
Aspergillus insuetus	10.48	2.48E+04	22.50	6.03E+04	0.61	2.24E+05	3.45	4.51E+04	25.00	2.31E+04	-	-	-	-	-	-	-	-	3.78E+05
Aspergillus tubingensis	19.05	4.51E+04	14.17	3.80E+04	43.64	1.62E+05	6.90	9.03E+04	-	-	5.13	4.66E+04	1.72	2.43E+03	2.63	2.47E+03	-	-	3.87E+05
Aureobasidium 01	-	-	-	-	1.82	6.73E+03	-	-	-	-	-	-	-	-	-	-	-	-	6.73E+03
Aureobasidium 02	0.95	2.26E+04	4.17	1.12E+04	1.21	4.49E+03	-	-	-	-	-	-	-	-	-	-	-	-	3.82E+04
Basifimbria sp.	-	-	-	-	-	-	-	-	-	-	2.56	2.33E+05	-	-	-	-	-	-	2.33E+05
Cephalotrichum microsporum	-	-	-	-	-	-	-	-	-	-	2.56	2.33E+04	-	-	-	-	-	-	2.33E+04
Chrysosporium 02	-	-	-	-	-	-	-	-	-	-	5.13	4.66E+04	-	-	-	-	-	-	4.66E+04
Cladosporium cladosporioides	0.95	2.26E+03	0.83	2.23E+03	0.61	2.24E+03	-	-	-	-	-	-	-	-	-	-	-	-	6.74E+03
Cladosporium herbarum	1.90	4.51E+03	0.83	2.23E+03	-	-	-	-	-	-	2.56	2.33E+07	-	-	-	-	-	-	2.33E+07
Cladosporium (ungrouped)	-	-	-	-	-	-	-	-	-	-	-	-	-	-	2.63	2.47E+04	-	-	2.47E+04
Clonostachys rosea	-	-	-	-	-	-	-	-	-	-	-	-	1.72	2.43E+03	-	-	3.85	2.53E+03	4.96E+03
Cylindrocarpon sp.	-	-	-	-	0.61	2.24E+03	-	-	-	-	-	-	-	-	-	-	-	-	2.24E+03
Fusarium 01	0.95	2.26E+03	-	-	-	-	-	-	-	-	-	-	-	-	2.63	2.47E+04	-	-	2.69E+04
Fusarium 02	-	-	-	-	-	-	-	-	-	-	-	-	1.72	2.43E+04	-	-	-	-	2.43E+04
Fusarium 03	-	-	-	-	-	-	-	-	-	-	-	-	1.72	2.43E+03	-	-	3.85	2.53E+03	4.96E+03
Fusarium 04	-	-	-	-	-	-	-	-	-	-	-	-	-	-	2.63	2.47E+03	3.85	2.53E+03	5.00E+03
Fusarium 05	-	-	0.83	2.23E+04	-	-	-	-	-	-	-	-	-	-	2.63	2.47E+04	-	-	4.70E+04

Taxon	M1		M2		M3		L1		L2		L3		N1		N2		N3		Total
	RA	CFU g^{-1}	RA	CFU g^{-1}	RA	CFU g^{-1}	RA	CFU g^{-1}	RA	CFU g^{-1}	RA	CFU g^{-1}	RA	CFU g^{-1}	RA	CFU g^{-1}	RA	CFU g^{-1}	CFU g^{-1}
Fusarium 08	-	-	-	-	-	-	-	-	-	-	-	-	1.72	2.43E+03	2.63	2.47E+03	3.85	2.53E+03	7.43E+03
Fusarium 09	-	-	-	-	-	-	-	-	-	-	-	-	1.72	2.43E+03	-	-	3.85	2.53E+03	4.96E+03
Fusarium 11	-	-	-	-	-	-	-	-	-	-	-	-	1.72	2.43E+03	5.26	4.93E+03	3.85	2.53E+03	9.89E+03
Fusarium 12	-	-	-	-	-	-	-	-	-	-	-	-	-	-	-	-	3.85	2.53E+03	2.53E+03
Fusarium 13	-	-	-	-	-	-	-	-	-	-	-	-	-	-	2.63	2.47E+03	-	-	2.47E+03
Fusarium 15	-	-	0.83	2.23E+04	-	-	-	-	-	-	-	-	1.72	2.43E+04	2.63	2.47E+03	3.85	2.53E+03	5.16E+04
Fusarium oxysporum	-	-	-	-	-	-	-	-	-	-	-	-	-	-	-	-	3.85	2.53E+03	2.53E+03
Geotrichum 01	-	-	-	-	-	-	-	-	-	-	-	-	-	-	-	-	3.85	2.53E+03	2.53E+03
Geotrichum 02	0.95	2.26E+05	-	-	-	-	-	-	-	-	-	-	-	-	-	-	-	-	2.26E+05
Humicola sp.	-	-	-	-	-	-	-	-	-	-	-	-	-	-	2.63	2.47E+03	-	-	2.47E+03
Marquandomyces marquandii	-	-	-	-	-	-	1.72	2.26E+04	-	-	2.56	2.33E+05	15.52	2.19E+04	5.26	4.93E+03	7.69	5.06E+03	2.88E+05
Microascus paisii	0.95	2.26E+04	0.83	2.23E+03	-	-	-	-	-	-	2.56	2.33E+04	-	-	-	-	-	-	4.81E+04
Penicillium charlesii	-	-	10.83	2.91E+04	-	-	1.72	2.26E+05	-	-	-	-	1.72	2.43E+03	-	-	-	-	2.57E+05
Penicillium citrinum	14.29	3.39E+04	27.50	7.37E+04	29.70	1.10E+05	43.10	5.64E+05	50.00	4.62E+04	12.82	1.17E+05	22.41	3.16E+04	13.16	1.23E+04	7.69	5.06E+03	9.94E+05
Penicillium estinogenum	-	-	-	-	-	-	-	-	-	-	-	-	8.62	1.22E+04	-	-	-	-	1.22E+04
Penicillium parvulum	-	-	0.83	2.23E+04	0.61	2.24E+04	3.45	4.51E+04	-	-	2.56	2.33E+05	-	-	-	-	-	-	3.23E+05
Penicillium pasqualense	12.38	2.93E+04	-	-	-	-	1.72	2.26E+05	-	-	2.56	2.33E+04	1.72	2.43E+03	2.63	2.47E+03	3.85	2.53E+03	2.86E+05
Penicillium scabrosum	9.52	2.26E+04	-	-	1.82	6.73E+04	1.72	2.26E+04	-	-	-	-	1.72	2.43E+03	-	-	7.69	5.06E+03	1.20E+05
Penicillium sizovae	-	-	-	-	-	-	1.72	2.26E+04	-	-	2.56	2.33E+04	-	-	-	-	-	-	4.59E+04
Penicillium skrjabinii	-	-	-	-	-	-	-	-	-	-	-	-	5.17	7.29E+03	2.63	2.47E+03	3.85	2.53E+03	1.23E+04
Penicillium soppii	-	-	-	-	-	-	-	-	-	-	-	-	1.72	2.43E+03	2.63	2.47E+03	3.85	2.53E+03	7.43E+03
Penicillium turbatum	0.95	2.26E+04	-	-	1.82	6.73E+04	12.07	1.58E+05	-	-	-	-	-	-	-	-	-	-	2.48E+05
Penicillium yarmokense	-	-	-	-	-	-	-	-	-	-	-	-	3.45	4.86E+03	2.63	2.47E+03	3.85	2.53E+03	9.86E+03
Penicillium (ungrouped)	-	-	0.83	2.23E+04	-	-	-	-	-	-	-	-	-	-	2.63	2.47E+03	-	-	2.48E+04
Pseudogymnoascus pannorum 1	6.67	1.58E+05	4.17	1.12E+05	-	-	3.45	4.51E+04	-	-	10.26	9.32E+04	1.72	2.43E+03	-	-	-	-	4.11E+05
Pseudo. pannorum 2	0.95	2.26E+04	0.83	2.23E+04	-	-	1.72	2.26E+05	-	-	2.56	2.33E+05	-	-	-	-	-	-	5.04E+05
Purpureocillium lilacinum	-	-	-	-	-	-	-	-	-	-	10.26	9.32E+04	1.72	2.43E+04	2.63	2.47E+03	-	-	1.20E+05
Pycnidia 01	-	-	-	-	-	-	-	-	-	-	-	-	3.45	4.86E+03	-	-	-	-	4.86E+03
Pycnidia 02	1.90	4.51E+04	-	-	-	-	-	-	-	-	-	-	-	-	-	-	-	-	4.51E+04
Pycnidia 05	-	-	0.83	2.23E+03	0.61	2.24E+03	-	-	-	-	-	-	-	-	-	-	-	-	4.48E+03
Scopulariopsis candida	-	-	-	-	-	-	-	-	-	-	-	-	-	-	-	-	-	-	0.00E+00
Talaromyces atricola	-	-	-	-	1.82	6.73E+04	-	-	-	-	-	-	-	-	-	-	-	-	6.73E+04
Talaromyces neorugulosus	-	-	-	-	-	-	-	-	-	-	2.56	2.33E+05	-	-	2.63	2.47E+03	-	-	2.36E+05
Trichoderma sp.	-	-	0.83	2.23E+04	12.73	4.71E+04	-	-	-	-	-	-	1.72	2.43E+03	13.16	1.23E+04	-	-	8.42E+04
Wardomyces sp.	-	-	-	-	-	-	-	-	-	-	-	-	1.72	2.43E+03	-	-	-	-	2.43E+03
Sterile 01	-	-	-	-	-	-	-	-	-	-	-	-	-	-	2.63	2.47E+03	-	-	2.47E+03
Sterile 03	0.95	2.26E+03	0.83	2.23E+03	-	-	-	-	-	-	-	-	-	-	-	-	-	-	4.49E+03
Sterile 07	-	-	-	-	-	-	-	-	-	-	-	-	1.72	2.43E+03	-	-	3.85	2.53E+03	4.96E+03
Sterile (ungrouped)	0.95	2.26E+04	0.83	2.23E+04	0.61	2.24E+04	-	-	-	-	2.56	2.33E+04	5.17	7.29E+03	2.63	2.47E+03	7.69	5.06E+03	1.05E+05

Taxon	M1		M2		M3		L1		L2		L3		N1		N2		N3		Total
	RA	CFU g^{-1}	RA	CFU g^{-1}	RA	CFU g^{-1}	RA	CFU g^{-1}	RA	CFU g^{-1}	RA	CFU g^{-1}	RA	CFU g^{-1}	RA	CFU g^{-1}	RA	CFU g^{-1}	CFU g^{-1}
Sporulating but undetermined	-	-	-	-	-	-	-	-	-	-	-	-	-	-	-	-	-	-	-
Total	100.0	**7.65E+05**	100.0	**8.72E+05**	100.0	**8.15E+05**	100.0	**1.92E+06**	100.0	**9.25E+04**	100.0	**2.55E+07**	100.0	2.72E+05	100.0	1.82E+05	100.0	6.58E+04	3.04E+07

Taxon	M1 RA	M1 CFU g^{-1}	M2 RA	M2 CFU g^{-1}	M3 RA	M3 CFU g^{-1}	L1 RA	L1 CFU g^{-1}	L2 RA	L2 CFU g^{-1}	L3 RA	L3 CFU g^{-1}	N1 RA	N1 CFU g^{-1}	N2 RA	N2 CFU g^{-1}	N3 RA	N3 CFU g^{-1}	Total CFU g^{-1}
Total Aspergillaceae	81.9	2.35E+05	82.5	4.02E+05	79.4	6.58E+05	91.4	1.60E+06	100.0	9.25E+04	51.3	6.76E+05	51.7	1.17E+05	34.2	3.21E+04	38.5	2.53E+04	3.84E+06
Total *Aspergillus*	44.8	1.26E+05	42.5	2.55E+05	45.5	3.91E+05	25.9	3.39E+05	50.0	4.62E+04	30.8	2.80E+05	5.2	5.10E+04	7.9	7.40E+03	7.7	5.06E+03	1.50E+06
Total *Penicillium*	37.1	1.08E+05	40.0	1.47E+05	33.9	2.67E+05	65.5	1.26E+06	50.0	4.62E+04	20.5	3.96E+05	46.6	6.56E+04	26.3	2.47E+04	30.8	2.03E+04	2.34E+06
Total *Talaromyces*	-	-	-	-	1.8	6.73E+04	-	-	-	-	2.6	2.33E+05	-	-	2.6	2.47E+03	-	-	3.03E+05
Total *Pseudogymnoascus*	7.6	1.81E+05	5.0	1.34E+05	-	-	5.2	2.71E+05	-	-	12.8	3.26E+05	1.7	2.43E+03	-	-	-	-	9.14E+05
Total Chaetothyriales	-	-	-	-	-	-	-	-	-	-	-	-	-	-	-	-	-	-	-
Total Cladosporiales	2.9	6.77E+03	1.7	4.47E+03	0.6	2.24E+03	-	-	-	-	2.6	2.33E+07	-	-	2.6	2.47E+04	-	-	2.33E+07
Total Hypocreales	1.0	2.26E+03	2.5	6.70E+04	13.9	5.16E+04	3.4	4.51E+04			15.4	5.59E+05	34.5	1.36E+05	52.6	1.16E+05	46.2	3.04E+04	1.01E+06
Total Microascales	1.0	2.26E+04	0.8	2.23E+03	-	-	-	-	-	-	5.1	4.66E+04	1.7	2.43E+03	-	-	-	-	7.39E+04
Total Onygenales	-	-	-	-	-	-	-	-	-	-	5.1	4.66E+04	-	-	-	-	-	-	4.66E+04
Total sterile and undetermined	3.8	7.00E+04	2.5	2.68E+04	1.2	2.47E+04	-	-	-	-	2.6	2.33E+04	12.1	1.70E+04	5.3	4.93E+03	11.5	7.59E+03	1.74E+05

Table S3. Relative abundance (RA) and colony forming units per gram of soil (CFU g^{-1}) of Fungi recovered on Malt Extract agar containing 20% sucrose (MEA20S) from soils from the tops of mound nests (M), within mound nests (L), and adjacent non-mound (N) sites.

Taxon	M1		M2		M3		L1		L2		L3		N1		N2		N3		Total
	RA	CFU g^{-1}	RA	CFU g^{-1}	RA	CFU g^{-1}	RA	CFU g^{-1}	RA	CFU g^{-1}	RA	CFU g^{-1}	RA	CFU g^{-1}	RA	CFU g^{-1}	RA	CFU g^{-1}	CFU g^{-1}
Acremonium 01	-	-	-	-	-	-	-	-	-	-	-	-	-	-	2.78	2.47E+03	-	-	2.47E+03
Acremonium 03	-	-	-	-	-	-	-	-	-	-	-	-	2.38	2.43E+05	-	-	-	-	2.43E+05
Acremonium 04	-	-	-	-	-	-	-	-	-	-	-	-	-	-	-	-	4.00	2.53E+03	2.53E+03
Acremonium 05	-	-	-	-	-	-	-	-	-	-	-	-	2.38	2.43E+04	2.78	2.47E+04	-	-	4.90E+04
Acremonium 06	-	-	-	-	-	-	-	-	-	-	-	-	-	-	2.78	2.00E+03	-	-	2.00E+03
Acremonium 11	1.69	2.26E+04	-	-	1.82	2.24E+04	-	-	-	-	-	-	-	-	-	-	-	-	4.50E+04
Acremonium 14	-	-	-	-	-	-	2.27	2.26E+05	4.00	2.31E+05	-	-	2.38	2.43E+03	2.78	2.47E+03	-	-	4.62E+05
Acremonium 15	-	-	-	-	-	-	-	-	4.00	2.31E+04	-	-	-	-	-	-	-	-	2.31E+04
Albifimbria verrucaria	1.69	2.26E+04	-	-	-	-	-	-	-	-	-	-	-	-	-	-	-	-	2.26E+04
Alternaria (ungrouped)	1.69	2.26E+04	3.45	2.23E+05	1.82	2.24E+04	-	-	-	-	-	-	-	-	-	-	-	-	2.68E+05
Aspergillus fructus	5.08	6.77E+04	6.90	4.47E+04	-	-	15.91	1.58E+05	16.00	9.25E+04	8.33	2.33E+05	2.38	2.43E+03	8.33	7.40E+03	-	-	6.06E+05
Aspergillus insuetus	1.69	2.26E+04	3.45	2.23E+04	1.82	2.24E+04	2.27	2.26E+05	4.00	2.31E+05	-	-	-	-	-	-	-	-	5.24E+05
Aspergillus tubingensis	6.78	9.03E+04	10.34	6.70E+04	5.45	6.73E+04	2.27	2.26E+04	-	-	-	-	-	-	-	-	-	-	2.47E+05
Aureobasidium 01	-	-	-	-	-	-	-	-	-	-	-	-	2.38	2.43E+03	-	-	-	-	2.43E+03
Aureobasidium 02	1.69	2.26E+04	6.90	4.47E+04	1.82	2.24E+04	-	-	-	-	-	-	-	-	-	-	-	-	8.97E+04
Cadophora fastigiata	-	-	-	-	-	-	-	-	-	-	-	-	-	-	-	-	4.00	2.53E+03	2.53E+03
Cephalotrichum microsporum	-	-	3.45	2.23E+04	-	-	-	-	4.00	2.31E+05	8.33	2.33E+05	-	-	-	-	-	-	4.87E+05
Chrysosporium 01	1.69	2.26E+04	-	-	-	-	-	-	-	-	-	-	-	-	-	-	-	-	2.26E+04

Taxon	M1		M2		M3		L1		L2		L3		N1		N2		N3		Total
	RA	CFU g^{-1}	RA	CFU g^{-1}	RA	CFU g^{-1}	RA	CFU g^{-1}	RA	CFU g^{-1}	RA	CFU g^{-1}	RA	CFU g^{-1}	RA	CFU g^{-1}	RA	CFU g^{-1}	CFU g^{-1}
Chrysosporium 02	-	-	-	-	-	-	-	-	-	-	8.33	2.33E+05	-	-	-	-	-	-	2.33E+05
Cladosporium cladosporioides	1.69	2.26E+04	3.45	2.23E+06	1.82	2.24E+04	-	-	-	-	-	-	2.38	2.43E+03	2.78	2.47E+05	-	-	2.53E+06
Cladosporium herbarum	3.39	4.51E+04	3.45	2.23E+04	1.82	2.24E+04	-	-	-	-	-	-	-	-	2.78	2.47E+05	-	-	3.37E+05
Cladosporium cf. *macrocarpum*	-	-	-	-	1.82	2.24E+04	-	-	-	-	-	-	-	-	2.78	2.47E+03	-	-	2.49E+04
Cladosporium cf. *sphaerospermum*	-	-	3.45	2.23E+05	50.91	6.29E+06	-	-	-	-	-	-	-	-	2.78	2.47E+05	-	-	6.76E+06
Clonostachys rosea	-	-	-	-	-	-	2.27	2.26E+04	-	-	-	-	-	-	2.78	2.47E+03	4.00	2.53E+03	2.76E+04
Dactylaria sp.	1.69	2.26E+04	-	-	-	-	-	-	-	-	-	-	-	-	-	-	-	-	2.26E+04
Exophiala sp.	-	-	-	-	-	-	-	-	-	-	-	-	-	-	-	-	4.00	2.53E+03	2.53E+03
Fusarium 08	-	-	-	-	-	-	-	-	-	-	-	-	2.38	2.43E+04	2.78	2.47E+04	4.00	2.53E+03	5.15E+04
Fusarium 09	-	-	-	-	-	-	-	-	-	-	-	-	2.38	2.43E+03	2.78	2.47E+03	-	-	4.90E+03
Fusarium 10	-	-	3.45	2.23E+04	-	-	-	-	-	-	-	-	-	-	-	-	-	-	2.23E+04
Fusarium 11	-	-	-	-	-	-	-	-	-	-	-	-	-	-	-	-	4.00	2.53E+03	2.53E+03
Fusarium 12	-	-	-	-	-	-	-	-	-	-	-	-	-	-	-	-	4.00	2.53E+03	2.53E+03
Fusarium 14	-	-	-	-	-	-	-	-	-	-	-	-	-	-	-	-	4.00	2.53E+03	2.53E+03
Fusarium 15	-	-	-	-	-	-	-	-	-	-	-	-	-	-	-	-	4.00	2.53E+03	2.53E+03
Gliocladium sp.	-	-	3.45	2.23E+04	-	-	-	-	-	-	-	-	-	-	-	-	-	-	2.23E+04
Humicola fuscoatra	-	-	-	-	-	-	-	-	-	-	-	-	2.38	2.43E+03	-	-	-	-	2.43E+03
Marquandomyces marquandii	-	-	3.45	2.23E+04	-	-	-	-	8.00	4.62E+04	16.67	4.66E+05	14.29	1.46E+04	2.78	2.47E+03	8.00	5.06E+03	5.57E+05
Microascus paisii	1.69	2.26E+04	3.45	2.23E+04	-	-	2.27	2.26E+05	-	-	-	-	-	-	-	-	-	-	2.71E+05
Penicillium charlesii	-	-	10.34	6.70E+04	1.82	2.24E+04	2.27	2.26E+04	4.00	2.31E+05	8.33	2.33E+05	-	-	-	-	-	-	5.76E+05
Penicillium chrysogenum	32.20	4.29E+06	-	-	-	-	-	-	-	-	-	-	-	-	-	-	-	-	4.29E+06
Penicillium citrinum	6.78	9.03E+04	6.90	4.47E+04	3.64	4.49E+04	56.82	5.64E+05	24.00	1.39E+05	16.67	4.66E+05	7.14	7.29E+03	2.78	2.47E+03	4.00	2.53E+04	1.38E+06
Penicillium parvulum	-	-	-	-	9.09	1.12E+06	-	-	-	-	-	-	2.38	2.43E+05	-	-	-	-	1.37E+06
Penicillium pasqualense	-	-	-	-	-	-	-	-	4.00	2.31E+04	-	-	19.05	1.94E+04	19.44	1.73E+04	4.00	2.53E+03	6.24E+04
Penicillium scabrosum	1.69	2.26E+04	-	-	-	-	-	-	16.00	9.25E+04	-	-	4.76	4.86E+03	2.78	2.47E+04	12.00	7.59E+03	1.52E+05
Penicillium sizovae	-	-	-	-	-	-	2.27	2.26E+04	-	-	8.33	2.33E+05	-	-	-	-	-	-	2.56E+05
Penicillium skrjabinii	-	-	-	-	-	-	-	-	-	-	-	-	9.52	9.72E+03	2.78	2.47E+03	-	-	1.22E+04
Penicillium soppii	-	-	-	-	-	-	-	-	-	-	-	-	2.38	2.43E+03	2.78	2.47E+03	4.00	2.53E+03	7.43E+03
Penicillium turbatum	1.69	2.26E+04	-	-	5.45	6.73E+04	9.09	9.03E+04	-	-	-	-	-	-	-	-	-	-	1.80E+05
Penicillium yarmokense	-	-	-	-	-	-	-	-	-	-	-	-	2.38	2.43E+03	-	-	-	-	2.43E+03
Penicillium (ungrouped)	1.69	2.26E+05	-	-	1.82	2.24E+04	-	-	-	-	-	-	2.38	2.43E+03	2.78	2.47E+05	4.00	2.53E+03	5.00E+05
Pseudogymnoascus pannorum 1	6.78	9.03E+04	6.90	4.47E+04	1.82	2.24E+04	2.27	2.26E+05	4.00	2.31E+05	16.67	4.66E+05	2.38	2.43E+04	2.78	2.47E+04	4.00	2.53E+03	1.13E+06
Pseudo. pannorum 2	1.69	2.26E+04	3.45	2.23E+04	-	-	-	-	8.00	4.62E+04	-	-	-	-	2.78	2.47E+04	-	-	1.16E+05
Purpureocillium lilacinum	1.69	2.26E+04	-	-	-	-	-	-	-	-	8.33	2.33E+05	2.38	2.43E+03	2.78	2.47E+04	4.00	2.53E+03	2.85E+05
Pycnidia 01	1.69	2.26E+04	-	-	1.82	2.24E+04	-	-	-	-	-	-	2.38	2.43E+03	-	-	4.00	2.53E+03	5.00E+04
Pycnidia 04	-	-	3.45	2.23E+04	-	-	-	-	-	-	-	-	-	-	-	-	-	-	2.23E+04
Pycnidia 05	1.69	2.26E+04	-	-	1.82	2.24E+04	-	-	-	-	-	-	-	-	-	-	-	-	4.50E+04
Talaromyces atricola	1.69	2.26E+04	-	-	-	-	-	-	-	-	-	-	2.38	2.43E+03	-	-	-	-	2.50E+04
Talaromyces neorugulosus	-	-	3.45	2.23E+04	-	-	-	-	-	-	-	-	-	-	-	-	-	-	2.23E+04

Taxon	M1		M2		M3		L1		L2		L3		N1		N2		N3		Total
	RA	CFU g^{-1}	RA	CFU g^{-1}	RA	CFU g^{-1}	RA	CFU g^{-1}	RA	CFU g^{-1}	RA	CFU g^{-1}	RA	CFU g^{-1}	RA	CFU g^{-1}	RA	CFU g^{-1}	CFU g^{-1}
Trichoderma sp.	1.69	2.26E+04	-	-	-	-	-	-	-	-	-	-	-	-	2.78	2.47E+04	-	-	4.72E+04
Trichosporiella cerebriformis	-	-	-	-	-	-	-	-	-	-	-	-	-	-	2.78	2.47E+03	-	-	2.47E+03
Sterile 01	-	-	-	-	-	-	-	-	-	-	-	-	-	-	5.56	4.93E+04	4.00	2.53E+03	5.19E+04
Sterile 04	1.69	2.26E+04	-	-	-	-	-	-	-	-	-	-	-	-	-	-	-	-	2.26E+04
Sterile 05	-	-	3.45	2.23E+05	-	-	-	-	-	-	-	-	-	-	-	-	-	-	2.23E+05
Sterile 07	1.69	2.26E+04	-	-	1.82	2.24E+04	-	-	-	-	-	-	2.38	2.43E+03	-	-	-	-	4.75E+04
Sterile (ungrouped)	5.08	6.77E+04	3.45	2.23E+04	1.82	2.24E+04	-	-	-	-	-	-	2.38	2.43E+03	5.56	4.93E+03	12.00	7.59E+03	1.27E+05
Sporulating but undetermined	-	-	-	-	-	-	-	-	-	-	-	-	-	-	-	-	-	-	-
Total	100.0	**5.40E+06**	100.0	**3.46E+06**	100.0	**7.90E+06**	100.0	1.81E+06	100.0	1.62E+06	100.0	2.80E+06	100.0	6.49E+05	100.0	1.26E+06	100.0	8.61E+04	2.50E+07

Total Aspergillaceae	57.6	4.83E+06	37.9	2.46E+05	29.1	1.37E+06	90.9	1.11E+06	68.0	8.09E+05	41.7	1.17E+06	52.4	2.94E+05	41.7	3.03E+05	28.0	4.05E+04	1.02E+07
Total *Aspergillus*	13.6	1.81E+05	20.7	1.34E+05	7.3	8.98E+04	20.5	4.06E+05	20.0	3.24E+05	8.3	2.33E+05	2.4	2.43E+03	8.3	7.40E+03			1.38E+06
Total *Penicillium*	44.1	4.65E+06	17.2	1.12E+05	21.8	1.28E+06	70.5	7.00E+05	48.0	4.86E+05	33.3	9.32E+05	50.0	2.92E+05	33.3	2.96E+05	28.0	4.05E+04	8.79E+06
Total *Talaromyces*	1.7	2.26E+04	3.4	2.23E+04	-	-	-	-	-	-	-	-	2.4	2.43E+03	-	-	-	-	4.73E+04
Total *Pseudogymnoascus*	8.5	1.13E+05	10.3	6.70E+04	1.8	2.24E+04	2.3	2.26E+05	12.0	2.77E+05	16.7	4.66E+05	2.4	2.43E+04	5.6	4.93E+04	4.0	2.53E+03	1.25E+06
Total Chaetothyriales	-	-	-	-	-	-	-	-	-	-	-	-	-	-	-	-	4.0	2.53E+03	2.53E+03
Total Cladosporiales	5.1	6.77E+04	10.3	2.48E+06	56.4	6.35E+06	-	-	-	-	-	-	2.4	2.43E+03	11.1	7.42E+05	-	-	9.65E+06
Total Hypocreales	6.8	9.03E+04	10.3	6.70E+04	1.8	2.24E+04	4.5	2.48E+05	16.0	3.01E+05	25.0	6.99E+05	28.6	3.13E+05	27.8	1.13E+05	40.0	2.53E+04	1.88E+06
Total Microascales	1.7	2.26E+04	6.9	4.47E+04	-	-	2.3	2.26E+05	4.0	2.31E+05	8.3	2.33E+05	-	-	-	-	-	-	7.57E+05
Total Onygenales	1.7	2.26E+04	-	-	-	-	-	-	-	-	8.3	2.33E+05	-	-	-	-	-	-	2.56E+05
Total sterile and undetermined	11.9	1.58E+05	10.3	2.68E+05	7.3	8.98E+04	-	-	-	-	-	-	7.1	7.29E+03	11.1	5.43E+04	20.0	1.27E+04	5.90E+05
